# Analysis and generation of motion profiles from positive and negative human actions

**DOI:** 10.3389/frai.2026.1963524

**Published:** 2026-10-06

**Authors:** Alessio Russo, Alessio Saccuti, Eleonora Iotti, Giuseppe Di Cesare

**Affiliations:** 1 Department of Food and Drug, University of Parma, Parma, Italy; 2 Italian Institute of Technology, Cognitive Architecture for Collaborative Technologies, Genova, Italy; 3 Department of Mathematical, Physical and Computer Sciences, University of Parma, Parma, Italy

**Keywords:** affective computing, deep learning, expressive motion analysis and synthesis, human motion analysis, vitality forms

## Abstract

Human actions convey not only their functional goal, the “what,” but also the manner in which they are performed, the “how.” The same action may be executed in a *Positive* or *Negative* manner depending on the agent's affective state. For example, handing someone an object carefully and politely reflects a positive manner, whereas doing so abruptly and dismissively reflects a negative one. This action modulation, known as Vitality Form (VF), is fundamental to human communication. Despite their importance, VFs remain relatively underexplored. This study investigates the kinematic signatures of *Positive* and *Negative* VFs in a pointing action and examines whether they can be reproduced through synthetic motion profiles. To this aim, kinematic data were collected from 40 right-handed participants performing three different pointing actions after recalling pleasant or unpleasant personal academic experiences. Each movement was assigned to either the *Positive* or *Negative* VF according to the emotional valence of the recalled experience. Several classifiers were evaluated for predicting VFs from wrist kinematic profiles, achieving a best accuracy of 73.97%. Grad-CAM analysis further provided insights into the kinematic basis of VF discrimination. We then investigated whether the kinematic patterns distinguishing *Positive* and *Negative* VFs could also be reproduced in synthetic motion profiles using generative models, achieving a Train-on-Synthetic, Test-on-Real accuracy of 72.50%. Finally, a perceptual study assessed whether the generated velocity profiles conveyed the intended *Positive* or *Negative* VFs. Participants identified the corresponding affective category with an accuracy of 75.80%, suggesting that affective information can be transferred to synthetic profiles and retained in a form that remains perceptually recognizable. Overall, this work advances our understanding of how affective states are encoded in human movement, demonstrating that these cues can be modeled, reproduced, and recognized in artificially generated actions. These findings have potential applications in neuroscience and social robotics, enabling artificial agents to recognize human attitudes and adapt their behavior accordingly.

## 1 Introduction

Human movements are never purely geometric. Two human actions may accomplish the same functional objective (the “what”), yet differ profoundly in the manner in which they are performed (the “how”). Unlike classical approaches on movement analysis, we focus on the latter aspect, the “how,” that includes qualitative modulation commonly referred to as *Vitality Form* (VF) (Di Cesare et al., 2020; Stern, 2010). VFs reflect the actor's affective state or communicative attitude through modulations of movement kinematics, while preserving the underlying action goal. VFs constitute key component for non-verbal communication. Indeed, they are fundamental to social interactions, as they convey critical information regarding an individual's attitude and underlying intentions. Although actions can be characterized by many different VFs, in this work we focus them into two broad categories: *Positive* and *Negative*. For example, passing an object gently may convey a positive attitude, whereas passing it abruptly may convey a negative one. In both cases, the functional goal is identical, while the form of execution differs.

The study of expressive qualities in human movement has been extensively investigated across psychology, neuroscience and affective computing (Stern, 2010; Di Cesare et al., 2020; Castellano et al., 2008; Garello et al., 2021). Several studies have focused on extracting affective and social information from human motion. Early motion analysis approaches relied on handcrafted kinematic descriptors, such as velocity, acceleration and smoothness (Flash and Hogan, 1985). More recently, Machine Learning (ML) and Deep Learning (DL) approaches have been employed to infer emotional states and social cues from human movement data (Castellano et al., 2008; Alotaibi and Solaiman, 2025). Recent advances have further explored the use of foundation models for the generation and modulation of expressive motion behaviors, demonstrating their potential to capture high-level movement characteristics and synthesize expressive human motion (Hielscher et al., 2026).

A related line of research, distinct from the VF-based approach proposed in this work and developed by other researchers, builds on the Laban Effort System, which is part of Laban Movement Analysis (LMA), a taxonomy originally developed for dance and theater training (Laban, 1964). The system describes movement along four bipolar factors: Space (Direct/Indirect), Weight (Strong/Light), Time (Sudden/Sustained), and Flow (Bound/Free), as detailed in Bernardet et al. (2019). Compared with VFs, Laban Efforts offer a more fine-grained, multi-axis decomposition of movement quality and have seen wider adoption in Human–Robot Interaction (HRI). For example, Knight and Simmons (2014) used Laban Effort features to communicate a robot's internal state through mobile-platform motion. The same framework was later applied to expressive head movements and to the modulation of functional task motions with superimposed Effort qualities (Knight and Simmons, 2015, 2016). Similarly, LaViers and Egerstedt (2012) formalized Laban-based style parameters for generating expressive robot dance. A somewhat different direction was explored by Rett et al. (2010), who used low-level features derived from LMA within a Bayesian classifier for HRI.

These works show that, unlike VFs, Laban Efforts have mainly been used to generate or modulate robot motion, while comparatively less attention has been devoted to recognizing expressive qualities from observed human behavior and transferring them to robot actions. Some recent works partially address this gap. Li et al. (2025) classify Light–Strong catching and releasing actions from EEG signals using a hybrid CNN–BiLSTM–Attention model, demonstrating recognition capability but requiring dedicated sensing hardware and addressing only classification. Nwagwu et al. (2026) introduce a dataset of 219 robot-arm demonstrations organized around the four Laban Effort axes and compare engineered-feature and sequential classifiers; however, the motions are intentionally elicited through physical guidance and no generative model is developed. In contrast, Raghu et al. (2025) generate hesitant robot motions by manually combining predefined Laban Shape and Effort parameters, showing that expressive qualities can be encoded in robot trajectories but without learning them automatically from human demonstrations. Overall, these studies address complementary aspects of expressive motion analysis, yet they either rely on specialized sensing or experimentally elicited behaviors, or use manually designed motion parameters. This leaves open the problem of recognizing expressive qualities directly from observable human motion and reproducing them through data-driven generation.

Regarding the latter aspect, generative modeling provides a natural framework for addressing this challenge. Generative approaches such as Variational Autoencoders (VAEs) (Kingma and Welling, 2013), Generative Adversarial Networks (GANs) (Goodfellow et al., 2014), and diffusion-based models (Ho et al., 2020) have been successfully applied to tasks including animation and robotic motion generation (Alemi et al., 2017; Tulyakov et al., 2018). Existing studies have also explored the synthesis of expressive movements (Garello et al., 2021; Ma et al., 2025; Dragan et al., 2013). However, these approaches are typically trained on datasets collected under experimental protocols in which participants are given explicit instructions to express specific emotions or movement styles. As a result, the generated motions may reflect instructed behaviors rather than naturally emerging movements driven by the individual's underlying affective state.

In contrast with the previously mentioned methodologies, in this work we propose a novel paradigm to investigate how human affective states are transformed into action kinematics. Rather than instructing participants how to mechanically perform the action, we elicit affective states through the recall of personally experienced academic events and analyze the resulting variations of movement execution in terms of VFs. In this preliminary study, we restrict the analysis to two broad affective conditions, *Positive* and *Negative*. To this end, we investigate whether VFs considered in this study affect action modulation and if the resulting kinematic information can subsequently be exploited to generate new actions with specific expressive characteristics. DL techniques are employed as methodological tools to carry out the analysis, classification, and generation tasks.

Models were trained on a dataset collected from a heterogeneous group of 40 right-handed volunteers. Each participant was asked to recall past experiences related to a specific affective state. Following each recall, participants performed a kinematic action, i.e., pointing at a target with their finger (example in Figure 1). To induce the desired VFs, we adopted a protocol previously used in the literature, as described by Di Cesare et al. (2025). Participants were asked to recall a positive academic experience (e.g., receiving a high grade on an exam) before executing the *Positive* action. Conversely, regarding the *Negative* action, a negative academic experience (e.g., unfairly failing an exam) was recalled.

**Figure 1:**
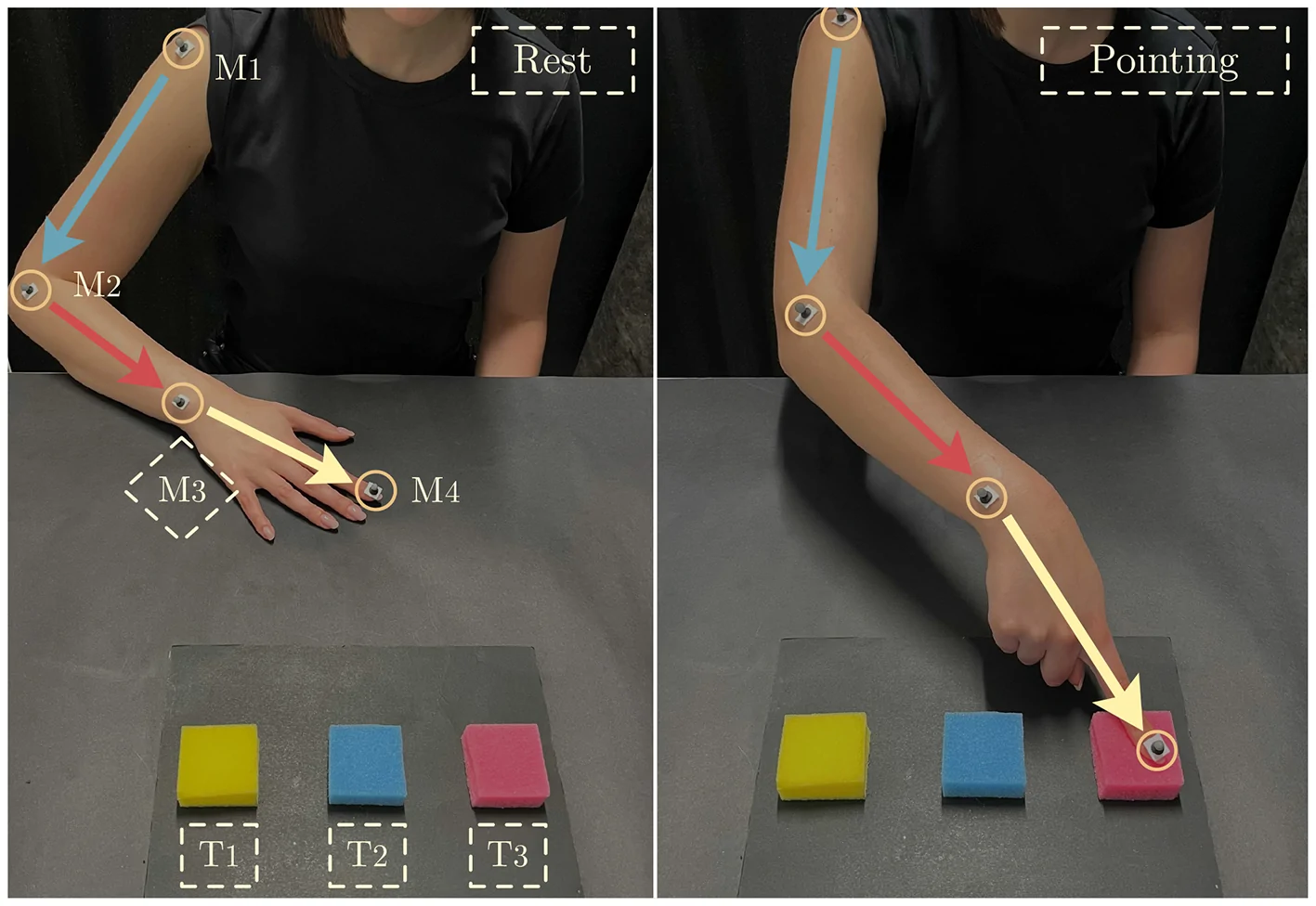
Experimental setup and kinematic chain used during data acquisition. The figure shows the starting (*Rest*) and final position (*Pointing*), as well as four markers placed on the shoulder (*m*_1_), elbow (*m*_2_), wrist (*m*_3_), and finger (*m*_4_), which were tracked using a motion capture system. The targets consist of three colored sponges, labeled *T*_1_, *T*_2_, and *T*_3_. Although all markers were recorded during data acquisition, the analyses presented in this paper are performed using only the wrist marker.

For the classification task, we compared DL-based strategies that take velocity, acceleration and jerk profiles as input. Our analysis focuses on hand motion profiles. For the sake of clarity, we define a *motion profile* as a description of how an object's motion changes over time, expressed either in terms of position, velocity, acceleration, or jerk.

Although focusing solely on hand motion limits the amount of captured body movement, this choice simplifies the experimental setup and facilitates deployment in constrained contexts, such as fMRI environments. This is particularly relevant for investigating the relationship between VFs and brain activity. Undeniably, the wrist is not the only body part capable of conveying information about VFs. Rather, VFs may be expressed through multiple components of human movement, and investigating their respective contributions lies beyond the scope of the present study. For instance, the same data could be collected using lightweight wearable sensors.

The output of each classifier corresponds to the *Positive* or *Negative* VF condition associated with the action. Specifically, we evaluated well-known approaches including the Autoregressive Integrated Moving Average model (ARIMA, Box et al., 2015), Long Short-Term Memory (LSTM, Hochreiter and Schmidhuber, 1997), and Gated Recurrent Unit (GRU, Cho et al., 2014), alongside a one-dimensional Convolutional Neural Network (1D-CNN, LeCun et al., 1998). The comparison showed that the best classification performance was achieved using the 1D-CNN-based DL approach.

Regarding the generative task, we defined and compared several DL solutions sharing the feature extraction backbone that achieved the best performance in the classification task. Each model takes motion profile velocity features as input and generates a corresponding synthetic velocity profile. We evaluated a Conditional Variational Autoencoder (CVAE, Sohn et al., 2015), a Conditional Generative Adversarial Network (GAN, Goodfellow et al., 2014), a Conditional Wasserstein Generative Adversarial Network (CWGAN, Arjovsky et al., 2017) and a Conditional Vector-Quantized Variational Autoencoder (CVQ-VAE, Van Den Oord et al., 2017). Among these, the CVQ-VAE emerged as the model that better approximates the human motion profiles.

In addition, we evaluated whether the synthetic velocity profiles produced by the CVQ-VAE preserve *Positive* and *Negative* VFs. We presented videos in which a 2D point moves along a curve, with motion consistent with the synthetic velocity profiles generated by the CVQ-VAE, and collected feedback on the perceived rudeness and gentleness.

In summary, the contributions are:

1. We designed a DL solution to classify hand motion profiles associated with *Positive* and *Negative* VFs, as elicited through the experimental affective manipulation, and compared it with existing baselines (Sections 2.1 and 3.2).
2. We developed DL-based generative models to synthesize motion profiles and evaluated them both quantitatively and qualitatively (Sections 2.2 and 3.3).
3. We provided evidence that the synthetic motion profiles preserve VF-related characteristics by showing videos to newly recruited participants. In each video, a 2D point moves along a Bézier curve with a velocity profile defined by the generated motion profiles (Section 3.4).

The rest of the paper is organized as follows. Section 2 presents the proposed methodology for the classification and generation of motion profiles. Section 3 describes the experimental setup and reports the classification, generation, and perceptual evaluation results. Finally, Section 4 discusses the main findings, practical implications, and limitations of the study.

## 2 Materials and methods

Given the set of markers {*m*_1_, *m*_2_, *m*_3_, *m*_4_} shown in Figure 1, we denote by $p_{m_{i}}\left(t\right)\in {\mathbb{R}}^{3}$ the three-dimensional position of marker *m*_*i*_ at time *t*∈ℝ_≥0_. The velocity, acceleration and jerk of the marker are then defined as $v_{m_{i}}\left(t\right)=\frac{d}{dt}p_{m_{i}}\left(t\right)$, $a_{m_{i}}\left(t\right)=\frac{d^{2}}{dt^{2}}p_{m_{i}}\left(t\right)$ and $j_{m_{i}}\left(t\right)=\frac{d^{3}}{dt^{3}}p_{m_{i}}\left(t\right)$, respectively.

As introduced in Section 1, we focus on hand motion profiles extracted from human actions. Accordingly, we define a motion profile as a one-dimensional signal describing the temporal evolution of a kinematic quantity associated with marker *m*_3_. Motion profiles can be categorized according to the kinematic quantity they represent. Specifically, velocity-, acceleration-, and jerk-bas*ed motion profiles correspond to the temporal evolution of the velocity, acceleration, and jerk norms, denoted by *s*_*v*_(*t*), *s*_*a*_(*t*), and *s*_*j*_(*t*), respectively.


$$\begin{array}{l} s_{v}\left(t\right)=||v_{m_{3}}\left(t\right)||,s_{a}\left(t\right)=||a_{m_{3}}\left(t\right)||,s_{j}\left(t\right)=||j_{m_{3}}\left(t\right)|| \end{array}$$ (1)

Motion profiles in Equation 1 are preprocessed to reduce noise and comply with the DL techniques used in this paper, which require each instance be of the same length. First, a standard moving-average filter was applied to suppress high-frequency noise. Subsequently, each smoothed time series was resampled to a fixed length using cubic spline interpolation (De Boor, 1978; Virtanen et al., 2020).

The resampled discrete motion profiles, denoted by ${\bar{s}}_{v}$, ${\bar{s}}_{a}$, and ${\bar{s}}_{j}$ for velocity-, acceleration-, and jerk-based representations, respectively, were associated with a binary label *y*∈0, 1 according to the affective condition used to elicit the corresponding VF, with *y* = 0 representing the *Negative* condition and *y* = 1 the *Positive* one. These labeled motion profiles were subsequently used to train DL models for classifying motion profiles according to the corresponding VF condition (Section 2.1) and for synthesizing new motion profiles that preserve VF-related characteristics (Section 2.2).

### 2.1 Classification of motion profiles under *positive* and *negative* VFs

Developing a classifier is a crucial component of this work, as it allows us to assess the separability of the proposed affective classes and determine whether emotional valence influences human movement in distinguishable ways. It also enables the use of Explainable Artificial Intelligence (XAI) to investigate how affective conditions shape motion profiles, as described in Section 3.2, and supports the evaluation of the motion profile generation method presented in Section 2.2.

Classifying motion profiles associated with *Positive* and *Negative* VFs using DL requires extracting discriminative features from the gestures and modeling their relationship with the affective condition used to elicit them. We compared several classification approaches, including ARIMA, GRU, LSTM, and a 1D-CNN. The latter, whose architecture is illustrated in Figure 2, constitutes the primary methodological contribution of this paper for modeling the relationship between human actions and VFs.

**Figure 2:**
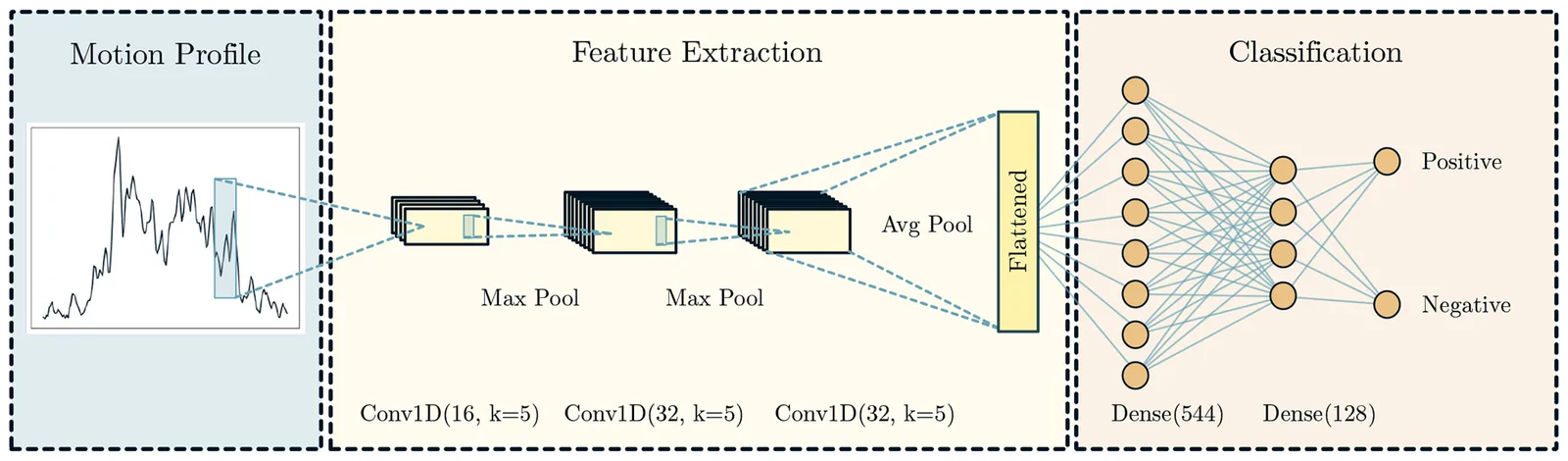
Architecture of the proposed 1D-CNN for VFs classification.

The choice of a convolutional architecture is motivated by its ability to capture local temporal dependencies and relevant kinematic patterns, such as abrupt peaks, smooth transitions, and variations in motion intensity, which are known to contribute to the perception of expressive movement (Castellano et al., 2008; Flash and Hogan, 1985).

The convolutional backbone is composed of three successive blocks. The first two blocks consist of a one-dimensional convolutional layer, followed by a Rectified Linear Unit (ReLU) (Nair and Hinton, 2010) activation and a max-pooling operation. The third block employs the same convolutional configuration, followed by an average-pooling layer. The resulting feature tensor is flattened into a latent representation, which is then processed by a two-layer Multi-Layer Perceptron (MLP) classification head. The network outputs a scalar logit ŷ, which is converted into the predicted probability of the *Positive* class through a sigmoid activation. The probability of the *Negative* class is then obtained as the complementary probability, 1−σ(ŷ).

The models were trained separately using ${\bar{s}}_{v}$, ${\bar{s}}_{a}$, and ${\bar{s}}_{j}$ to evaluate the influence of velocity-, acceleration- and jerk-based inputs on classification performance. Model training was conducted using the fenn library.^1^ Binary Cross-Entropy was used as the loss function, while AdamW (Loshchilov and Hutter, 2017) was employed for optimization.

### 2.2 Generation of motion profiles

Motion generation models should generate profiles that retain the structural and dynamic characteristics of human movement. The extent to which these properties are preserved depends on the model architecture and on how motion features are extracted and represented. To investigate form preservation and compare established approaches with alternative design choices, we implemented several standard deep learning models based on VAEs and GANs, including a CVAE, a CGAN and a CWGAN. In addition, we propose a CVQ-VAE as a contribution of this work.

The use of a CVQ-VAE is motivated by its ability to better preserve the variability of observed motion patterns and thus maintaining human-like properties. While VAEs and GANs may struggle to represent the full structure of the data, the discrete latent representation induced by vector quantization allows the CVQ-VAE to encode distinct motion patterns more effectively, making it better suited to capture both variability and structure.

Figure 3 illustrates the CVQ-VAE architecture. The model takes as input a velocity-based motion profile ${\bar{s}}_{v}$ and it is designed to synthesize new profiles with either *Positive* or *Negative* VFs. Although acceleration and jerk information were available (Equation 1), we chose to use only velocity-based motion profiles. This choice enables the synthesized profiles to be directly used for the modulation of Bézier trajectories (Section 3.4). Conversely, acceleration and jerk norms do not preserve the direction of motion and therefore cannot be used for this purpose.

**Figure 3:**
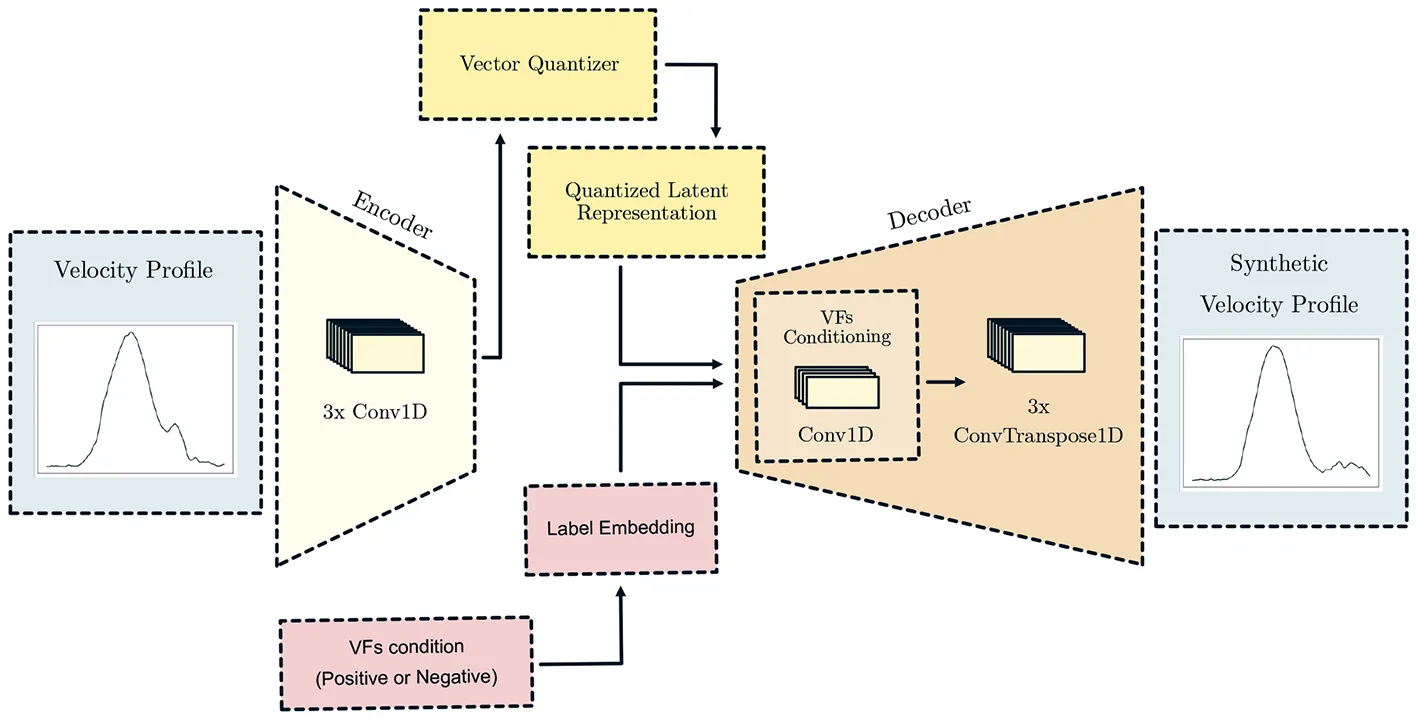
Architecture of the proposed CVQ-VAE for VF-conditioned hand velocity profile generation.

The encoder follows the same convolutional backbone introduced in Section 2.1. This design choice is motivated by the results obtained in the classification task (Section 3.2), where convolutional models proved effective in capturing local temporal dependencies and expressive kinematic patterns from wrist motion signals.

After encoding the input sequence into a compact latent representation, the model introduces a vector quantization (VQ) bottleneck, which replaces each latent vector with the closest element from a learned codebook (a collection of patterns). This operation transforms the continuous latent representation into a discrete sequence of prototype vectors, encouraging the model to represent motion as a composition of a finite set of recurring patterns.

The decoder incorporates VFs conditioning by repeating a learned class label embedding along the temporal dimension and concatenating it with the quantized latent representation. A dedicated convolutional layer then mixes the latent features and the conditioning signal while preserving temporal resolution.

The decoder uses a hierarchy of three transposed 1D convolutional layers to reconstruct the motion profile, symmetrically mirroring the encoder's structure. Each layer performs temporal upsampling, progressively increasing the sequence length while reducing feature channels. ReLU activations follow the intermediate layers to add expressivity. The final transposed convolution maps the features back to the original number of input channels, producing the reconstructed velocity profile with output padding configured to ensure exact reconstruction of the original sequence length.

We trained the CVQ-VAE by minimizing a joint loss function composed of a reconstruction term, and a vector-quantization term. Optimization is performed using AdamW (Loshchilov and Hutter, 2017).

## 3 Results

This section presents the results of our classification and generation experiments. We begin by describing the data acquisition process and the resulting dataset (Section 3.1), followed by a quantitative and qualitative performance analysis of the proposed models (respectively, Sections 3.2 and 3.3). Finally, we evaluate whether the synthesized motion profiles preserve perceptually distinguishable forms through a human perception study (Section 3.4).

### 3.1 Experimental data acquisition

Forty right-handed volunteers, recruited in academic contexts, participated in the experiment. To avoid possible bias in the experimental procedure, we ensured that none of the participants reported a history of psychiatric or neurological disorders or current use of psychoactive medications. Moreover, each participant was asked to sign an informed consent form stating that the study was approved by the Local Ethics Committee of Parma (Prot.[CB2.1] 41-2026-N) in accordance with the Declaration of Helsinki.

Experimental data were collected by asking each participant to perform two tasks: inducing and maintaining a specific affective state previously experienced in real life (feeling task, *FEEL*), and subsequently executing a pointing action (execution task, *EXE*). In particular, each pointing action could be performed toward one of three possible targets (*T*_1_, *T*_2_, or *T*_3_), as shown in Figure 1. During the *FEEL* task, participants were given 15 s to induce a positive (e.g., the feeling of passing an exam with a high grade) or negative (e.g., the feeling of unfairly failing an exam) affective state.

After this feeling period, participants transferred this affective state into the corresponding pointing action by modulating the motion profile (the *EXE* task).

The experiment consisted of a positive and a negative experimental session, each lasting 5 min, separated by a brief break. The positions of the four markers were tracked throughout each session using an OptiTrack Trio system, and labels were assigned based on the corresponding session type.

Each participant repeated the pointing action nine times for each affective state condition, resulting in an initial total of 720 trials. After excluding seven trials due to acquisition noise, the final dataset comprised 713 samples, with 359 actions labeled as *Positive* and 354 labeled as *Negative*. This resulted in a substantially balanced class distribution for the subsequent analysis.

### 3.2 Classification performance on real motion profiles

To verify the capability of the model proposed in Section 2.1 to capture the form of an action while ensuring its ability to generalize across subjects, we adopted a *subject-wise five fold cross-validation* (Kohavi, 1995). In each fold, data from 32 subjects were used for training, while the remaining eight subjects were held out for testing. This evaluation strategy prevents information leakage between the training and test sets and therefore provides a more reliable estimate of the model's robustness.

The proposed 1D-CNN classifier was compared against three baseline models commonly employed for temporal sequence modeling, namely ARIMA, LSTM, and GRU. Performance was evaluated in terms of classification accuracy (%). Each model was trained three times, once for each type of motion profile (velocity-, acceleration-, and jerk-based), to assess whether the choice of kinematic representation influences model performance.

Table 1 reports the models' mean accuracy and standard deviation across the five folds for velocity-based (Velocity), acceleration-based (Acceleration), and jerk-based (Jerk) input representations. The best performance is achieved by the 1D-CNN using the wrist acceleration signal, with an average accuracy of 73.97%. In contrast, the recurrent baselines, particularly the LSTM and GRU models, perform close to chance level. These results are consistent with the hypothesis that discriminative information may be captured effectively by localized temporal patterns in the motion profiles.

**Table 1:** Classification accuracy (%) across different models.

Model	Velocity	Acceleration	Jerk
ARIMA	**66.66** **±7.96**	69.17 ± 6.26	63.81 ± 5.91
LSTM	50.07 ± 0.91	49.22 ± 1.53	52.35 ± 5.50
GRU	49.93 ± 0.91	49.23 ± 3.53	48.67 ± 1.79
1D-CNN	64.67 ± 6.46	**73.97** **±6.25**	**63.95** **±8.22**

Results are reported as mean ± standard deviation. Bold values indicate the highest classification accuracy obtained for each input feature.

To further investigate this hypothesis, we employed a state-of-the-art Explainable Artificial Intelligence (XAI) technique, Grad-CAM (Selvaraju et al., 2017), to qualitatively analyze the motion profiles. Specifically, the proposed 1D-CNN was used to classify acceleration-based motion profiles of pointing actions, and the resulting Grad-CAM activation maps were projected onto the temporal acceleration profiles, highlighting the portions of the motion that contributed most to the classifier's decision as either a *Positive* or *Negative* condition. We report four representative examples in Figure 4. The Grad-CAM visualizations reveal that the activations are predominantly concentrated around phases of high acceleration. These phases may occur either in the first part of the motion profile, corresponding to the subject accelerating during the pointing action (Figure 4C) or in the latter part, corresponding to the subject decelerating before reaching the target (Figure 4A). This finding indicates that the classifier relies primarily on localized temporal patterns rather than processing the entire motion profile uniformly. A comparison between Figures 4C, D further supports this observation. These examples correspond to *Positive* and *Negative* motion profiles, respectively. Although both profiles exhibit acceleration peaks of comparable magnitude, they are assigned to different classes. This suggests that the discriminative information associated with the VFs is not encoded by isolated kinematic descriptors, such as peak acceleration or peak velocity, but rather by the temporal context in which these features occur. Another noteworthy observation is that the classifier does not focus on a single acceleration phase. When a motion profile contains multiple regions of high acceleration with comparable intensity, the network attends to all of them, as illustrated in Figure 4B. This behavior is consistent with the hypothesis that the kinematic cues associated with *Positive* and *Negative* VFs are distributed across multiple informative temporal segments rather than concentrated in a single salient event. These observations also suggest that localized temporal patterns may be particularly informative for discriminating between the two conditions.

**Figure 4:**
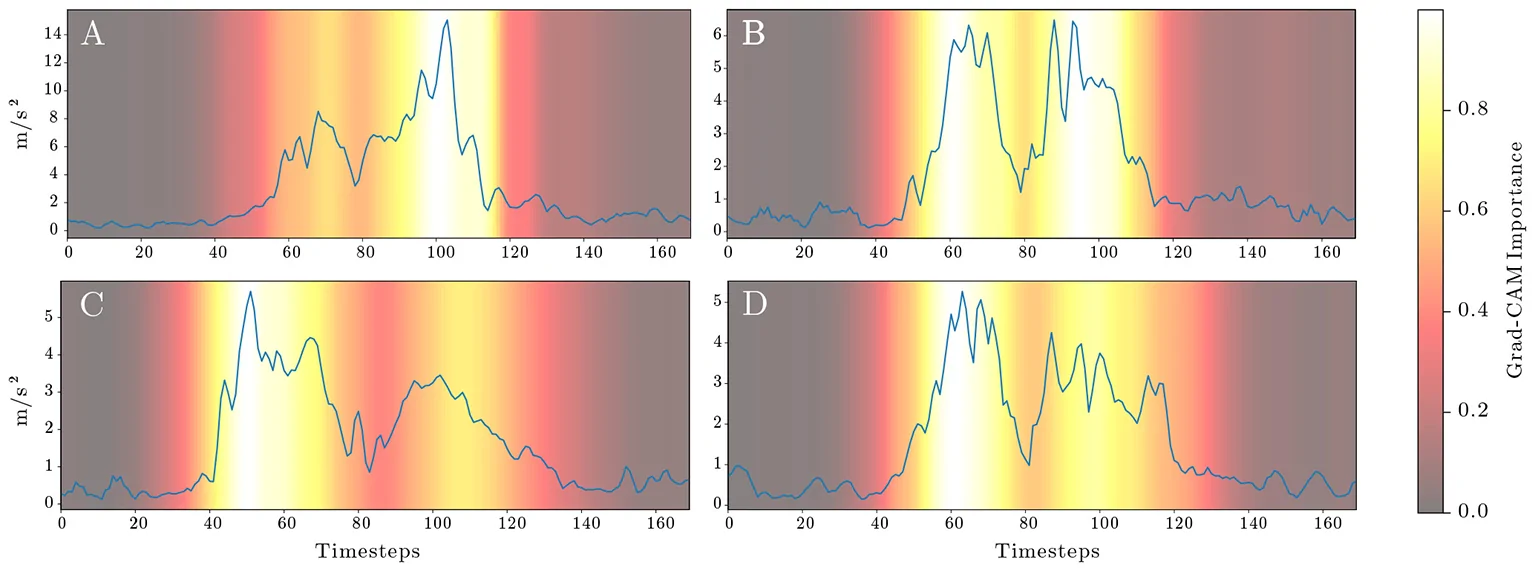
Grad-CAM activation maps for four representative acceleration profiles [(**A, C**) *Positive*, (**B, D**) *Negative*], highlighting the temporal regions that contribute most to the 1D-CNN classification.

### 3.3 Generation results

This section evaluates the ability of the proposed generative models to preserve kinematic characteristics associated with the VFs. We present a quantitative analysis based on cross-domain classification metrics (Section 3.3.1), followed by a qualitative assessment of the generated profiles' temporal structure, variability, and similarity to real human trajectories. In particular, we first compare the global distribution of real and synthetic motion profiles (Section 3.3.2). Secondly, we assessed whether the shape of original samples is preserved in synthetic motion profiles (Section 3.3.3).

#### 3.3.1 Classification performance on synthetic motion profiles

To evaluate the quality of the generated velocity profiles, we conducted a cross-classification analysis between real and synthetic samples. Because Section 3.2 demonstrated that acceleration-based representations provide the most discriminative information for distinguishing the VFs, we evaluated the generative models in this feature space by computing the acceleration of the synthetic wrist velocity profiles.

Specifically, synthetic acceleration signals were computed by differentiating the generated velocity profiles at the OptiTrack sampling frequency of 120 Hz, followed by taking the absolute value to match the non-negative nature of the original data, as shown in Equation 1.

To determine whether synthetic samples preserve the discriminative properties associated with the VFs, we used the 1D-CNN proposed in Section 2.1 to compute the accuracy in two standard settings for synthetic data validation, namely: *Train on Real data, Test on Synthetic data* (TRTS) and *Train on Synthetic data, Test on Real data* (TSTR) (Esteban et al., 2017; Garello et al., 2021). Results for the CGAN, CWGAN, CVAE, and CVQ-VAE models are reported in Table 2.

**Table 2:** Cross-domain classification accuracy (%) using the TRTS and TSTR evaluation metrics.

Metric	CGAN	CWGAN	CVAE	CVQ-VAE
TRTS	36.19	44.29	**73.07**	65.44
TSTR	55.71	54.72	64.17	**72.50**

Bold values indicate the highest accuracy obtained for each evaluation metric (TRTS and TSTR).

The CGAN and CWGAN exhibited similarly poor performance in both settings, achieving a maximum TSTR accuracy of only 55.71%. This indicates a limited capacity to preserve the VF-related discriminative characteristics of real gestures. In contrast, both the CVAE and CVQ-VAE achieve substantially stronger results, highlighting their suitability for synthetic gesture generation. The CVAE achieves the highest TRTS accuracy of 73.07%, suggesting a strong alignment between its synthetic samples and the discriminative patterns found in real acceleration signals. Meanwhile, the CVQ-VAE reaches the highest TSTR accuracy of 72.50%, indicating that classifiers trained on its synthetic samples generalize effectively to real-world data.

Overall, results in Table 2 indicate that both VAE-based approaches are promising, though further analysis is required to identify which model provides the most faithful representation of the underlying signal characteristics.

#### 3.3.2 Qualitative distributional analysis of generated motion profiles

To further investigate the ability of the selected models to preserve the structure of the original signals, we supplemented the quantitative TRTS and TSTR analysis with a qualitative evaluation of the generated kinematic profiles. The goal is to verify whether the synthetic trajectories retain the morphology of real gestures and to assess their overall diversity and variability.

In this section we compare the global distribution of both real and synthetic profiles through the mean velocity at each timestamp, together with the corresponding ±1σ envelope for each VFs condition, as shown in Figure 5.

**Figure 5:**
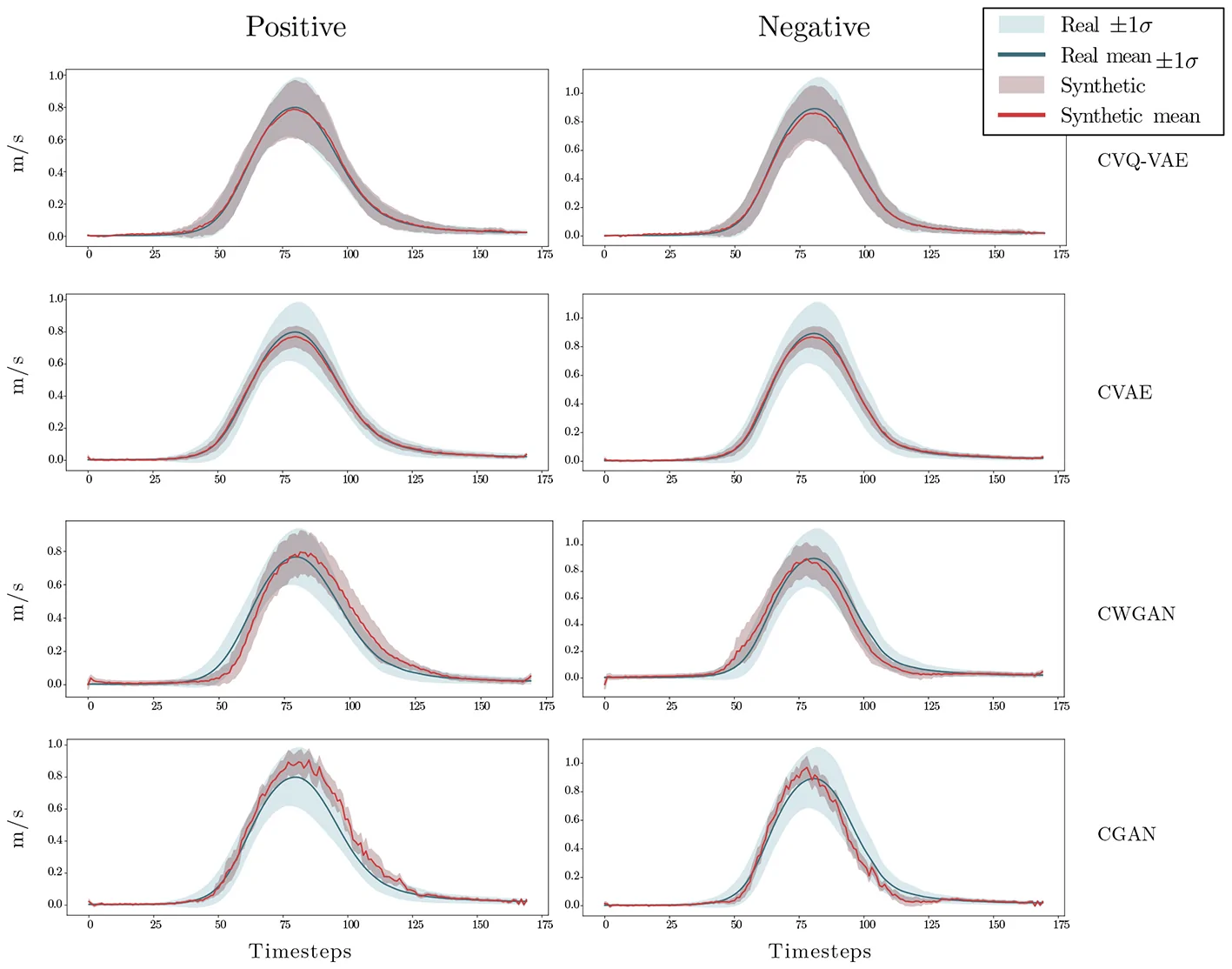
Comparison of real and synthetic velocity distributions for the two VFs across the evaluated generative models. For each class, the mean trajectory with its ±1σ envelope is shown. Rows correspond to CVQ-VAE, CVAE, WGAN and CGAN (**top** to **bottom**); columns correspond to the *Positive* **(left)** and *Negative* **(right)** classes.

The resulting curves confirm that the CGAN is unable to accurately reproduce either the average temporal behavior of the real samples or their variability. Specifically, the mean velocity of the generated *Negative* class is consistently lower than that of the real data, whereas the mean of the generated *Positive* class is generally higher. In both cases, the synthetic ±1σ envelope is considerably narrower than the real one, indicating limited diversity in the generated profiles. Furthermore, the trajectories produced by the CGAN appear noticeably noisier than the real data. While the real curves are smooth, the generated ones exhibit fluctuations in both the mean and the standard deviation at nearly every time step. The CWGAN improves the variability of the generated motion profiles compared with the CGAN, producing a standard deviation envelope that more closely resembles the real one. However, the generated trajectories remain noticeably noisy. In contrast, both the CVAE and the CVQ-VAE successfully capture the mean temporal behavior of the real data without introducing the high-frequency fluctuations observed in the GAN-based models. Compared with the CVQ-VAE, the CVAE exhibits a reduced ability to reproduce trajectory variability, as reflected by its narrower standard deviation envelope. This limitation may stem from the regularization imposed on the latent space, which constrains the learned representations toward a Gaussian distribution. By contrast, the discrete latent representation of the CVQ-VAE better preserves sample diversity, resulting in a variability profile that more closely matches that of the real data.

#### 3.3.3 Qualitative assessment of real and synthetic shape similarity

In this section we conducted a nearest-neighbor similarity analysis using cosine similarity to assess whether the shape of individual samples is preserved (Figure 6). For a subset of real samples, we identified the most similar trajectories within a synthetic dataset of equivalent size (713 samples: 359 *Positive* and 354 *Negative*) by maximizing the cosine similarity score (Equation 2).


$$\begin{array}{c} s\left(v_{\text{syn}},v_{\text{real}}\right)=\frac{v_{\text{syn}}^{⊤}v_{\text{real}}}{||v_{\text{syn}}{||}_{2}||v_{\text{real}}{||}_{2}}. \end{array}$$ (2)

**Figure 6:**
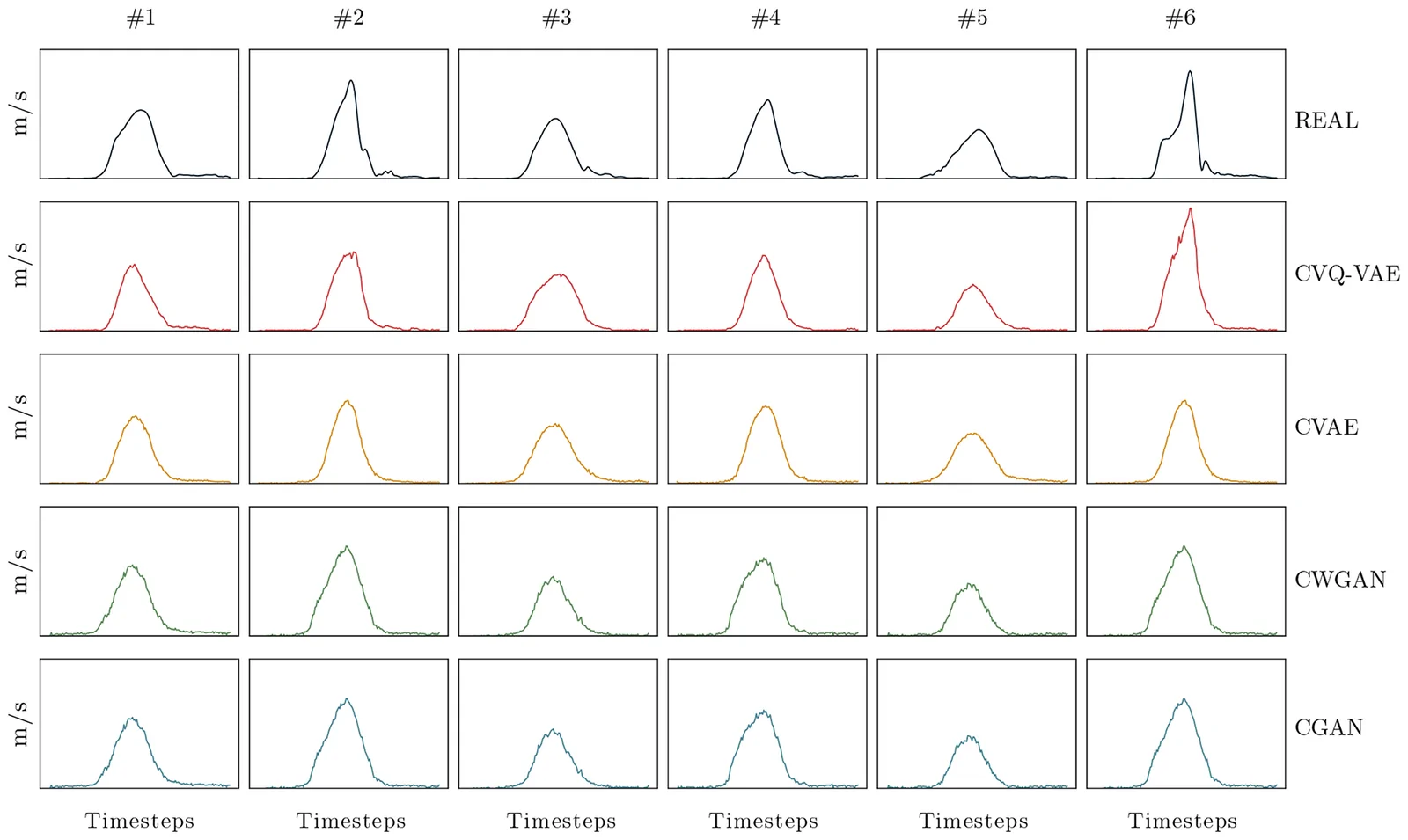
Qualitative nearest-neighbor comparison for the *Negative* VF condition. Each synthetic velocity motion profile generated by the CVQ-VAE, CVAE, WGAN, and GAN is paired with its most similar real velocity profile from the dataset **(top row)** according to cosine similarity. The strong correspondence in temporal morphology confirms the preservation of class-specific kinematic patterns.

Cosine similarity was selected as a shape-oriented metric because it emphasizes the similarity of temporal profiles independently of absolute amplitude scaling.

Figure 6 shows representative real velocity profiles and the most similar synthetic profile generated by each model. All models reproduce the overall bell-shaped structure of the pointing trajectories. Differences become more apparent for profiles that depart from a smooth and approximately symmetric bell shape. In addition, the CWGAN and CGAN profiles exhibit visibly higher local noise than those generated by the CVAE and CVQ-VAE, particularly along the rising and falling portions of the velocity peak and in the low-velocity regions.

In examples 2 and 6, the real trajectories contain sharp and asymmetric peaks. The CVQ-VAE reproduces these characteristics more closely, whereas the CVAE tends to generate smoother and more symmetric profiles. The CWGAN and CGAN also approximate the global peak structure, but their outputs are characterized by stronger high-frequency fluctuations. For more regular trajectories, such as those in examples 1, 4, and 5, the CVAE and CVQ-VAE provide qualitatively comparable approximations. The distinction is less evident in example 3. Although the real profile contains small fluctuations after the main peak, none of the models fully reproduces this fine-grained structure. The CWGAN and CGAN exhibit several local variations; however, these fluctuations are not consistently aligned with those of the real profile and therefore appear to reflect generation noise rather than faithful preservation of its temporal morphology. Overall, the figure suggests that the CVQ-VAE better preserves pronounced deviations from a regular bell-shaped trajectory, particularly peak asymmetry and sharpness, while also producing smoother profiles than the adversarial baselines.

This qualitative result is consistent with, although it does not directly demonstrate, the Grad-CAM findings in Figure 4. The Grad-CAM analysis indicates that classification of the VFs relies on localized regions of the acceleration profile. Accordingly, generative models that preserve localized changes in temporal morphology may retain discriminative information more effectively than models that reproduce only the global motion envelope. At the same time, the local fluctuations produced by the CWGAN and CGAN should not necessarily be interpreted as informative temporal detail, since they are not consistently matched to the morphology of the corresponding real profiles.

These observations may also contribute to the different TRTS and TSTR results. The comparatively regular profiles generated by the CVAE may be easier for a classifier trained on real data to recognize, potentially supporting its TRTS performance. Conversely, the closer reproduction of sharp and asymmetric profiles by the CVQ-VAE, without the pronounced local noise observed in the CWGAN and CGAN outputs, may expose a classifier to more realistic intra-class variation and could contribute to its stronger TSTR generalization. These interpretations should nevertheless be regarded as qualitative hypotheses rather than direct causal evidence.

### 3.4 Vitality forms perception

To evaluate the perception of the generated motion profiles, we conducted an anonymous study involving 45 newly recruited participants. Each participant was presented with 15 pairs of videos, with each pair consisting of one *Positive* and one *Negative* synthetic motion profile, presented in randomized order. Participants were asked to identify which video corresponded to the *Positive* and which one corresponded to the *Negative* condition. To produce the videos, we generated 30 motion profiles (15 *Positive* and 15 *Negative*) using the CVQ-VAE model and used the synthesized velocities to move a point along a given curve (the trajectory). In particular, to separately control the spatial geometry of the trajectory and its temporal evolution, the path shape is defined independently of the velocity profile.

Specifically, synthetic trajectories were created as quadratic Bézier curves (Farin, 2002), defined by a start point **p**_0_, an end point **p**_2_, and a control point **p**_1_ that determines the curvature. The point **p**_1_ is placed at the midpoint of the segment connecting **p**_0_ and **p**_2_, then displaced perpendicularly by an amount proportional to the Euclidean distance between the endpoints. This offset is regulated by a curvature parameter κ∈[−1, 1], which controls both the direction and magnitude of the deviation from a straight path. The curve is defined as:


$$\begin{array}{c} \text{B}\left(t\right)={\left(1-t\right)}^{2}{\text{p}}_{0}+2\left(1-t\right)t{\text{p}}_{1}+t^{2}{\text{p}}_{2},\;t\in \left[0,1\right]. \end{array}$$ (3)

The curve is then densely sampled to estimate its cumulative arc length (Farin, 2002), that is, the distance traveled along the path from the starting point. This representation allows to determine how far the motion progresses along the same curve over time by using velocity-based motion profile.

Given a synthesized velocity profile, the sequence is first rescaled so that its cumulative sum matches the total length of the curve. The cumulative distances obtained in this way identify the successive positions to be reached along the path. These distances are then converted into the corresponding Bézier parameters through interpolation, and the final trajectory is obtained by evaluating Equation 3 at those parameter values.

This construction ensures that the generated motion starts exactly at **p**_0_, ends at **p**_2_, and follows the temporal modulation imposed by the synthesized velocity profile, as shown in Figure 7. The resulting trajectories preserve the same spatial endpoints while modulating the geometric shape and temporal dynamics as a function of the imposed VFs condition.

**Figure 7:**
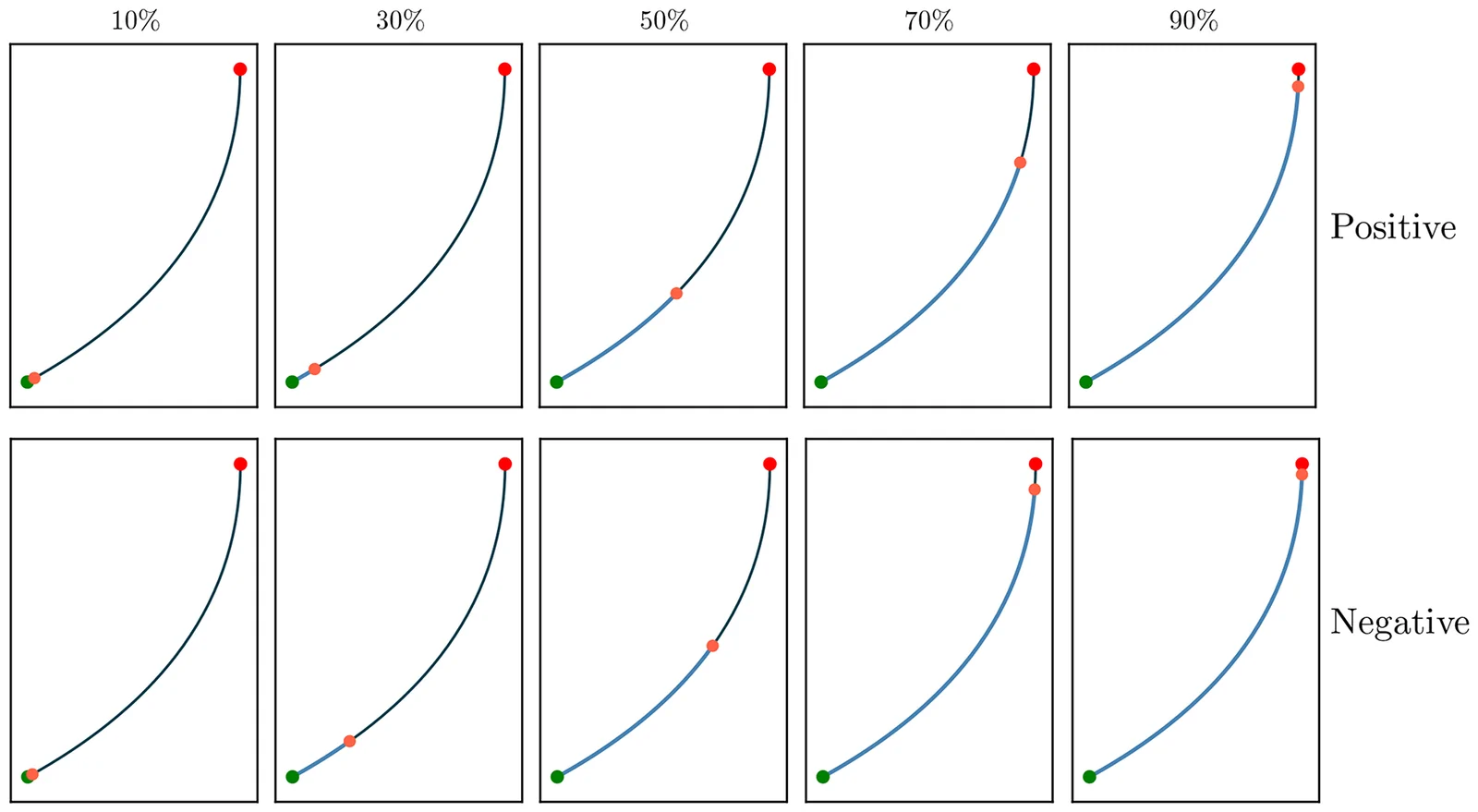
Examples of generated 2D trajectories. Each row shows five video frames: the first, of a trajectory generated under the *Positive* VF condition; the second, of a trajectory generated under the *Negative* one.

The perceptual experiment yielded a mean per-participant accuracy of 75.80% (SD = 19.74%), suggesting that the synthesized motion profiles conveyed distinguishable VF-related characteristics. In our experiment, the two VF classes, *Positive* and *Negative*, were presented in substantially equal proportions to each participant. Therefore, under the null hypothesis that participants' responses are independent of the VF conveyed by the motion, the expected accuracy is 50%. Importantly, this remains the expected accuracy even in the presence of a systematic response preference toward one of the two classes, because the two classes occur equally often. A one-sample *t*-test against the 50% chance level showed that mean participant-level performance was significantly above chance, *t*_(45)_ = 8.82, *p* < 0.001. Despite substantial inter-participant variability, these results indicate that, at the group level, participants' responses were systematically related to the VF conveyed by the synthesized motions, supporting the presence of perceptually distinguishable VF-related characteristics.

## 4 Discussion

The present study investigated whether *Positive* and *Negative* VFs can be identified from human movement kinematics and reproduced through DL-based generative models. Overall, the findings highlighted three main results, which represent the primary contributions of this work. *Firstly*, wrist motion profiles contained sufficient information to discriminate between *Positive* and *Negative* VFs. *Secondly*, deep generative models were able to synthesize new motion profiles while preserving the affective characteristics of the original human actions. *Thirdly*, the generated profiles remained perceptually distinguishable, demonstrating that the synthesized actions retained communicative properties recognizable by human observers.

One of the main findings concerns the classification of VFs. Among all evaluated models, the proposed 1D-CNN achieved the best performance while using acceleration motion profiles as input. This result is consistent with the hypothesis that the affective modulation of pointing actions may be encoded in localized temporal variations in movement dynamics rather than in long-range sequential dependencies. The Grad-CAM analysis further provides qualitative support for this interpretation by showing that the classifier focuses around phases of high acceleration, which may occur during either the subject's acceleration or deceleration phase. Notably, the network appeared not to rely solely on isolated kinematic descriptors, such as peak acceleration alone, but rather on the temporal organization surrounding these events. These findings are consistent with previous studies showing that expressive qualities of movement emerge from dynamic temporal structure rather than from individual kinematic parameters (Flash and Hogan, 1985; Castellano et al., 2008). The best classification accuracy reaches 73.97%, substantially outperforming the 50% chance level for the binary task. This result indicates that the proposed model successfully captures discriminative kinematic patterns associated with the two VFs, providing further evidence that the same action was executed with distinguishable expressive dynamics.

From a neuroscience perspective, these findings are particularly relevant because they provide a computational characterization of movement features associated with VFs. Previous neurophysiological studies demonstrated that the dorso-central insula is selectively involved in both the execution and perception of VFs, i.e., the affective quality of actions (Di Cesare et al., 2020, 2025). The present work extends these findings by identifying the kinematic information that may underlie such neural representations. Although our study does not directly investigate neural activity, the identified acceleration patterns may represent candidate computational features underlying the sensorimotor encoding of affective movement.

The generative findings further demonstrate that preserving the temporal structure of movement is essential for reproducing expressive human actions. GAN-based approaches struggled to preserve both the variability and temporal morphology observed in real movements. In contrast, both VAE-based approaches produced substantially more faithful reconstructions. Among them, the proposed CVQ-VAE achieved the best balance between realism and variability. The discrete latent representation introduced by vector quantization appears particularly suitable for representing recurrent motion primitives while simultaneously preserving natural inter-sample variability. This interpretation is supported by the quantitative TSTR evaluation (accuracy of 72.50%) and the qualitative analyses. In particular, the distribution and nearest-neighbor findings showed that the CVQ-VAE better maintained atypical trajectory shapes that were frequently smoothed by the conventional CVAE.

An important contribution of the present study is the perceptual validation of the generated motion profiles. Participants correctly identified the intended VF in the presented stimuli, with an accuracy of 75.80%, demonstrating that the synthesized kinematic patterns preserved communicative information that remained meaningful to human observers. This result suggests that the learned representations capture aspects of movement that are relevant not only statistically but also behaviorally. The analysis of misclassifications may also provide valuable insights and represents an interesting direction for future research.

The present findings also have practical implications. Generating human-like expressive movements remains a key challenge for social robotics and virtual agents, such as embodied conversational agents and digital avatars, where successful interaction depends not only on executing actions accurately but also on conveying appropriate interpersonal attitudes through movement. Previous research has shown that humans spontaneously perceive the VFs expressed through robotic movements (Di Cesare et al., 2020). The proposed framework may therefore provide a computational basis for enabling robots and virtual agents to both recognize and generate socially meaningful action dynamics. More broadly, the proposed methodology could contribute to a wide range of applications in which the natural modulation of movement quality plays an increasingly important role, including rehabilitation, education, and remote communication.

A few limitations should nevertheless be acknowledged. First, the dataset consisted of relatively simple pointing actions performed by healthy university students following an academic mood-induction procedure. Whether the proposed models generalize to more complex actions, spontaneous affective expressions, or different populations remains to be established. Second, only two affective conditions, Positive and Negative, were investigated. Given the self-induced affective manipulation adopted in this study, this binary setting was intentionally chosen as a controlled preliminary step before extending the paradigm to a broader range of affective states. These two contrasting VFs were selected to maximize their observable effects on the kinematic characteristics of the performed action. Moreover, because they represent familiar and readily recognizable ways of performing an action, participants could more easily evoke the corresponding state and translate it into movement. VFs are likely to span a richer multidimensional space, including subtle gradations of interpersonal attitudes that future work should address. Third, the kinematic analysis was restricted to the the wrist. Nevertheless, the dynamic features associated with VFs are unlikely to be expressed exclusively through this body segment. Other body regions may contribute to their communication, either independently or through coordinated whole-body behavior. Lower-limb actions, gait, postural adjustments, and global body dynamics can all influence how the manner of an action is perceived. For example, differences in walking rhythm or posture may make a person appear uncertain, determined, or energetic. Further research should therefore extend the analysis beyond wrist trajectories to establish whether VFs share common kinematic and neural mechanisms across body regions or whether their representation varies according to the effector involved.

Overall, the present study demonstrates that the affective quality of human actions can be extracted from movement kinematics, computationally modeled, and successfully transferred to synthetic motion profiles, retaining perceptually recognizable affective information associated with the original actions. These findings provide new insights into the computational representation of VFs while offering a promising framework for generating socially expressive artificial movements in neuroscience, HRI, and affective computing. In future work, this strategy will also be applied to real-world applications, for example, to modulate human actions for reproduction on humanoid robotic platforms such as the iCub (Metta et al., 2010).

## Data Availability

The raw data supporting the conclusions of this article will be made available by the authors upon request.

## Footnotes

1
https://github.com/pyfenn/fenn
